# Clinical Advances in the Diagnosis and Treatment of Biliary Tract Diseases

**DOI:** 10.3390/jcm12062282

**Published:** 2023-03-15

**Authors:** Saburo Matsubara

**Affiliations:** Department of Gastroenterology and Hepatology, Saitama Medical Center, Saitama Medical University, Saitama 350-8550, Japan; saburom@saitama-med.ac.jp; Tel.: +81-49-228-3400 (ext. 7839); Fax: +81-49-226-5284

Although the biliary tract is a small organ, it is diverse in location (intrahepatic bile ducts, extrahepatic bile ducts, gallbladder, and ampulla) and disease (including benign and malignant), thus a multidisciplinary approach involving endoscopists, surgeons, oncologists, radiologists, and infectious disease specialists is often required to treat biliary tract disease. In particular, obstructive jaundice and cholangitis associated with biliary stasis are common complications, and biliary drainage by endoscopic retrograde cholangiopancreatography (ERCP) is an indispensable procedure. In the past, percutaneous transhepatic biliary drainage (PTBD) has been performed in cases of difficult ERCP, such as surgical altered anatomy (SAA) or duodenal obstruction, but there have been many incidents and problems with external drainage, which reduce the patient’s quality of life. Recently, new endoscopic techniques such as balloon endoscopy-assisted ERCP (BE-ERCP) and endoscopic ultrasound-guided biliary drainage (EUS-BD) have been developed and are widely used as alternatives to PTBD and ERCP.

Among EUS-BD procedures, EUS-guided hepaticogastrostomy (EUS-HGS) has traditionally been performed only in high-volume centers because of its high technical difficulty and the possibility of serious complications such as biliary peritonitis and stent migration, but recent advances in devices and the accumulation of knowledge on techniques [1,2] have made it possible for trained endoscopists to perform EUS-HGS safely and successfully in general hospitals [3]. In the future, not only will the further development of dedicated devices but also education using phantom models will be important for generalization.

Both EUS-BD and BE-ERCP are useful in cases of SAA, but each procedure has its own advantages and disadvantages, and it is important to select the method according to the patient’s condition and the endoscopist’s experience [4]. Especially, BE-ERCP is a well-established treatment for common bile duct stones with SAA, with a high success rate and low incidence of complications [5].

For the management of combined malignant biliary and duodenal obstruction, double stenting is required. Traditionally, transpapillary biliary drainage with ERCP and duodenal stenting have been used, but the technical difficulty of transpapillary drainage and duodenobiliary reflux have been a problem. Recently, EUS-guided novel methods such as EUS-BD- and EUS-guided gastrojejunostomy (EUS-GJ) for duodenal drainage have been developed; moreover, new metal stents, such as an anti-reflux metal stent (ARMS) and lumen-apposing metal stent (LAMS), have been developed, which are expected to provide better treatment results [6].

The gold standard of treatment for acute cholecystitis is laparoscopic cholecystectomy, but gallbladder drainage is performed in patients with a high surgical risk. In recent years, many reports have demonstrated the usefulness of endoscopic gallbladder drainage including transpapillary drainage and EUS-guided drainage as alternatives to percutaneous transhepatic gallbladder drainage (PTGBD); however, the long-term preventive effect on recurrent cholecystitis has not yet been fully clarified [7]. Further studies are needed.

The current standard of care for unresectable hilar malignant biliary obstruction is bilateral drainage with an uncovered self-expandable metal stent (UCSEMS). However, recent advances in chemotherapy have prolonged survival and increased the chance of stent occlusion, and unremovable UCSEMS have become a problematic barrier to reintervention. A fully covered SEMS (FCSEMS) can be removed and is expected to have a good patency period due to its larger diameter compared to a plastic stent [8], but there is still little evidence, so future large-scale clinical trials are needed.

The standard treatment for ampullary tumors is pancreatoduodenectomy, but adenomas and carcinomas in situ can be cured by local resection without lymph node dissection. Local resection includes endoscopic papillectomy (EP) and surgically transduodenal ampullectomy, but the less invasive EP is considered the first choice when possible [9].

Although transpapillary drainage is the gold standard for the treatment of acute cholangitis, there is no settled opinion regarding the duration of antibiotics administration. Recently, shorter durations have been recommended to prevent the emergence of resistant organisms, shorten hospital stays, and reduce costs. For mild or moderate cholangitis, two days of antibiotics administration may be sufficient [10] if an appropriate drainage is performed. Transpapillary drainage was also shown to be safe and possibly effective for severe cholangitis with disseminated intravascular coagulation (DIC) [11].

In recent years, evidence regarding chemotherapy for biliary tract cancer has been increasing. In addition to conventional cytotoxic agents, many clinical trials have been conducted on molecularly targeted agents and immunotherapy [12]. As for adjuvant therapy, capecitabine is the standard of care in Western countries, while S-1 is the standard of care in Japan [13]. Genetic and molecular biological testing for personalized medicine [14] will become increasingly important in the future.

This Special Issue contains various articles on the recent advances in the clinical aspects of biliary tract diseases. Although the COVID-19 pandemic had a major impact on healthcare worldwide, it was possible to diagnose and treat biliary tract diseases without delay by taking appropriate exposure prevention measures [15]. It is hoped that the various difficulties that will befall us will be overcome, and that this field will make further progress in the future.

## References

[1] Matsubara S., Nakagawa K., Suda K., Otsuka T., Oka M., Nagoshi S. (2022). Practical Tips for Safe and Successful Endoscopic Ultrasound-Guided Hepaticogastrostomy: A State-of-the-Art Technical Review. J. Clin. Med..

[2] Kobori I., Hashimoto Y., Shibuki T., Okumura K., Sekine M., Miyagaki A., Sasaki Y., Takano Y., Katayama Y., Kuwada M. (2022). Safe Performance of Track Dilation and Bile Aspiration with ERCP Catheter in EUS-Guided Hepaticogastrostomy with Plastic Stents: A Retrospective Multicenter Study. J. Clin. Med..

[3] Kuraoka N., Hashimoto S., Matsui S., Terai S. (2021). Outcomes of Endoscopic Ultrasound-Guided Biliary Drainage in a General Hospital for Patients with Endoscopic Retrograde Cholangiopancreatography-Difficult Transpapillary Biliary Drainage. J. Clin. Med..

[4] Tanisaka Y., Mizuide M., Fujita A., Ogawa T., Suzuki M., Katsuda H., Saito Y., Miyaguchi K., Tashima T., Mashimo Y. (2021). Recent Advances of Interventional Endoscopic Retrograde Cholangiopancreatography and Endoscopic Ultrasound for Patients with Surgically Altered Anatomy. J. Clin. Med..

[5] Obata T., Tsutsumi K., Kato H., Ueki T., Miyamoto K., Yamazaki T., Matsumi A., Fujii Y., Matsumoto K., Horiguchi S. (2021). Balloon Enteroscopy-Assisted Endoscopic Retrograde Cholangiopancreatography for the Treatment of Common Bile Duct Stones in Patients with Roux-en-Y Gastrectomy: Outcomes and Factors Affecting Complete Stone Extraction. J. Clin. Med..

[6] Takeda T., Sasaki T., Okamoto T., Sasahira N. (2021). Endoscopic Double Stenting for the Management of Combined Malignant Biliary and Duodenal Obstruction. J. Clin. Med..

[7] Inoue T., Yoshida M., Suzuki Y., Kitano R., Okumura F., Naitoh I. (2021). Long-Term Outcomes of Endoscopic Gallbladder Drainage for Cholecystitis in Poor Surgical Candidates: An Updated Comprehensive Review. J. Clin. Med..

[8] Matsubara S., Nakagawa K., Suda K., Otsuka T., Oka M., Nagoshi S. (2022). The Feasibility of Whole-Liver Drainage with a Novel 8 mm Fully Covered Self-Expandable Metal Stent Possessing an Ultra-Slim Introducer for Malignant Hilar Biliary Obstructions. J. Clin. Med..

[9] Sekine M., Watanabe F., Ishii T., Miura T., Koito Y., Kashima H., Matsumoto K., Noda H., Rikiyama T., Mashima H. (2021). Investigation of the Indications for Endoscopic Papillectomy and Transduodenal Ampullectomy for Ampullary Tumors. J. Clin. Med..

[10] Masuda S., Koizumi K., Makazu M., Uojima H., Kubota J., Kimura K., Nishino T., Sumida C., Ichita C., Sasaki A. (2022). Antibiotic Administration within Two Days after Successful Endoscopic Retrograde Cholangiopancreatography Is Sufficient for Mild and Moderate Acute Cholangitis. J. Clin. Med..

[11] Sekine A., Nakahara K., Sato J., Michikawa Y., Suetani K., Morita R., Igarashi Y., Itoh F. (2021). Clinical Outcomes of Early Endoscopic Transpapillary Biliary Drainage for Acute Cholangitis Associated with Disseminated Intravascular Coagulation. J. Clin. Med..

[12] Sasaki T., Takeda T., Okamoto T., Ozaka M., Sasahira N. (2021). Chemotherapy for Biliary Tract Cancer in 2021. J. Clin. Med..

[13] Miyata Y., Kogure R., Nakazawa A., Nagata R., Mitsui T., Ninomiya R., Komagome M., Maki A., Kawarabayashi N., Beck Y. (2021). The Efficacy of S-1 as Adjuvant Chemotherapy for Resected Biliary Tract Carcinoma: A Propensity Score-Matching Analysis. J. Clin. Med..

[14] Perez-Moreno P., Riquelme I., Brebi P., Roa J.C. (2021). Role of lncRNAs in the Development of an Aggressive Phenotype in Gallbladder Cancer. J. Clin. Med..

[15] Ikemura M., Tomishima K., Ushio M., Takahashi S., Yamagata W., Takasaki Y., Suzuki A., Ito K., Ochiai K., Ishii S. (2021). Impact of the Coronavirus Disease-2019 Pandemic on Pancreaticobiliary Disease Detection and Treatment. J. Clin. Med..

